# Comprehensive transcriptomic analysis reveals turnip mosaic virus infection and its aphid vector *Myzus persicae* cause large changes in gene regulatory networks and co-transcription of alternative spliced mRNAs in *Arabidopsis thaliana*

**DOI:** 10.1186/s12870-024-06014-3

**Published:** 2025-01-30

**Authors:** Venura Herath, Clare L. Casteel, Jeanmarie Verchot

**Affiliations:** 1 Department of Agricultural Biology, Faculty of Agriculture, University of Peradeniya, Kandy, 20400 Sri Lanka; 2 Department of Plant Pathology and Plant-Microbe Biology, Cornell University, Ithaca, NY USA; 3 School of Integrative Plant Science, Section of Plant Pathology & Plant-Microbe Biology, Cornell University, Ithaca, NY USA; 4 Department of Plant Pathology & Microbiology, Texas A&M University, College Station, TX 77845 USA

**Keywords:** Potyvirus, Aphids, Hormone signaling, Transcriptomics

## Abstract

**Background:**

Virus infection and herbivory can alter the expression of stress-responsive genes in plants. This study employed high-throughput transcriptomic and alternative splicing analysis to understand the separate and combined impacts on host gene expression in *Arabidopsis thaliana* by *Myzus persicae* (green peach aphid), and turnip mosaic virus (TuMV).

**Results:**

By investigating changes in transcript abundance, the data shows that aphids feeding on virus infected plants intensify the number of differentially expressed stress responsive genes compared to challenge by individual stressors. This study presents evidence that the combination of virus-vector-host interactions induces significant changes in hormone and secondary metabolite biosynthesis, as well as downstream factors involved in feedback loops within hormone signaling pathways. This study also shows that gene expressions are regulated through alternative pre-mRNA splicing and the use of alternative transcription start and termination sites.

**Conclusions:**

These combined data suggest that complex genetic changes occur as plants adapt to the combined challenges posed by aphids and the viruses they vector. This study also provides more advanced analyses that could be used in the future to dissect the genetic mechanisms mediating tripartite interactions and inform future breeding programs.

**Supplementary Information:**

The online version contains supplementary material available at 10.1186/s12870-024-06014-3.

## Background

Many animal and plant infecting viruses have been known to elicit spatiotemporal changes in host gene expression in a manner that impairs the translation of cellular mRNA and host defenses to favor virus gene expression, known as host gene shut-off [1]. More than two decades ago, researchers showed that transient downregulation of host gene expression accompanies pea seed-borne mosaic virus (PSbMV; a potyvirus) replication in embryonic and cotyledon tissues [2] and the simultaneous increase in HSP70 (heat shock protein 70) and polyubiquitin expression [3]. Transcriptome data, obtained through RNAseq, has revolutionized our ability to assess gene expression patterns in response to various virus infections and insect feeding. Virus infection and aphid probing/feeding are known to regulate the expression of various plant signaling pathways involved in stress and immune responses. Recent scientific reports present new evidence that key viral proteins directly influence plant gene regulatory networks and, in some cases facilitate aphid infestation and transmission by modulating plant transcription factors, protein turnover pathways, and defense signaling pathways [4–6].

The genus *Potyvirus* provides a robust number of virus species known to manipulate host gene expression. Virus-encoded HC-Pro, VPg, NIa-Pro, NIb (RNA-dependent RNA polymerase), and 6K2 proteins directly influence host gene expression to create an environment favoring infection and herbivory [7–9]. HC-Pro for example, which is well-known for binding virions to aphid stylets for vector transmission, also binds to the RAV2 (related to ABI3/VP1 2) transcription factor to induce host stress and defense-related gene expression including factors that interfere with antiviral silencing [10]. In addition, the potyvirus HC-Pro and RAV2 combine to influence the expression of genes involved in responses to wounding, jasmonic acid (JA), cold, and heat stress [10]. The VPg-NIa-Pro blocks host translation by sequestering in the nucleus and nucleolus with the eIF4E (eukaryotic translation initiation factor 4E), S6k (ribosomal protein S6 kinase), and the PABP (poly(A) binding protein) [8, 11, 12]. The potyviral NIa-Pro increases aphid performance on infected *Arabidopsis thaliana* plants by inhibiting ethylene dependent plant defenses [4]. The NIb protein is known to recruit many cellular proteins including RNA helicase-like proteins AtRH8 and AtRH9, and NbEXPA1 to viral replication complexes in the cytoplasm, while in the nucleus it interacts with SCE1 (SUMO-conjugating enzyme 1) to block antiviral NPR1 (nonexpressor of PR genes 1) activities [7, 8]. Mutations disrupting the nuclear translocation of NIb abolish virus infection suggesting that NIb has some undiscovered abilities to influence host gene expression [7]. The TuMV 6K2 protein while embedded in the ER triggers the unfolded protein response (UPR) by activating three basic leucine-zipper (bZIP) transcription factors, bZIP60, bZIP28 and bZIP17, and increasing the expression of chaperones needed for proper protein folding [13, 14]. bZIP60 and bZIP28 combined influence virus titers in systemically infected plants.

While the vast majority of transcriptomic studies involving virus and insect challenges to plants have focused on changes in mRNA abundance [15, 16], new studies involving sugarcane mosaic virus (SCMV), potato virus Y (PVY), and bean common mosaic virus (BCMV) are noting the accumulation of different mRNA isoforms that are regulated through alternative splicing (AS) of pre-mRNAs [17–21]. For example, JAZ (jasmonate-zim-domain) proteins represent a group of transcriptional repressor proteins known to affect JA responses. AS of the *JAZ10* pre-mRNA generates 3 variants encoding proteins with different C-terminal domains which also differ in their sensitivity to rising jasmonic acid-isoleucine (JA-Ile) concentrations (JA)-induced degradation [22]. The *ZmPSY1* (*maize phytoene synthase 1*) gene encodes a key enzyme in carotenoid biosynthesis that is required for normal chloroplast function and is a pro-viral host factor influencing the pathogenesis of SCMV. Two splicing variants of *ZmPSY1* known as T001 and T003 only differ in the length of the 3’ UTR which is a region that controls its own mRNA translation activity. Research indicates that SCMV alters the splicing pattern to favor T001 transcript accumulation which plays a more significant role in promoting SCMV titer and disease while preserving chloroplast functions [20].

Despite the frequent co-occurrence of aphids and viruses in natural environments and synergistic interactions, the molecular mechanisms mediating these two agents together have received limited attention (see: [23–26]. Furthermore, only a few studies have investigated the role of AS in the regulation of plant–insect interactions. For example, a study of *Zea mays* (maize) under normal conditions and after aphid herbivory reported significant differences in gene expression and AS regulation [27]. Similarly, a transcriptomic study of *Manduca sexta* caterpillar feeding on *Nicotiana attenuata* leaves generated mostly distinct sets of differentially spliced genes and differentially expressed genes in the leaves and roots [28]. To address some of these gaps in knowledge, our study aims to expand the understanding of plant transcriptional response to stress using *Arabidopsis thaliana*, *Myzus persicae* (green peach aphid), and turnip mosaic virus (TuMV) as a model system. We reanalyzed our published RNA-seq experiments in new ways to generate novel hypotheses concerning the function of plant transcriptional responses to aphids, TuMV, and the dual challenge of TuMV and aphids. Furthermore, we identified isoforms in the RNA sequencing (RNA-seq) data, enabling the identification of unique and previously unexplored transcriptional responses. The research outcomes hold substantial importance in enhancing our understanding of plant defense mechanisms against the combined threats of aphids and viruses.

## Methods

### Plant material and aphid/virus treatments

Recently, we characterized the transcriptome of *Arabidopsis thaliana* plants with and without TuMV infection and aphid-infestation [6]. In this published work, wild-type *A. thaliana* ecotype Columbia-0 plants were obtained from the Arabidopsis Biological Resource Center and grown under controlled conditions of 25/20 °C day/night with a photoperiod of 14/10 h day/night, a relative humidity of 50%, and a light intensity of 200 mmol m − 2 s − 1. After three weeks of growth, half of the plants were rub-inoculated with TuMV:GFP, which was propagated from the infectious clone pCAMBIA:TuMV-GFP as in [29]. One week after infection fully infected leaves were identified by fluorescence under UV light, and 15 adult apterous aphids were caged on one leaf per plant for six uninfected and six infected plants. A corresponding set of six infected and six uninfected plants received cages with no aphids as controls for aphid feeding. Caged leaves were developmentally matched, and infected infection was verified before caging based on GFP visualization (Additional file 1). After 48 h of aphid placement, cages and aphids were removed, and leaves were pooled for every two plants resulting in replicates for each treatment.

## Transcriptomic analysis

Read quality was assessed using FastQC (v 0.11.9) and then the reference-guided mapping was carried out using the *A. thaliana* Col-0 genome assembly TAIR10 in The Arabidopsis Information Resource (TAIR) database (https://www.arabidopsis.org/index.jsp, last accessed 15.03.2022) using HISAT2 (v2.2.1) [30], a more advanced alignment program than in the previously reported analysis [6]. The workflow is illustrated in Additional file 1. The SAM files were converted to BAM files and indexed using SAMtools (v1.10) [31, 32]. Assembly alignment quality was assessed using FASTQC (v. 0.11.9). Transcripts assembly and abundance were determined using StringTie (v2.1.0) [33] and using the annotations obtained from Thalemine (v5.1.0–20221003). Raw sequence counts were calculated using HT-Seq (v2.0.1) (Putri et al., 2022). Overall, between 10.5 and 15.7 million raw reads were mapped to the reference Arabidopsis genome, and among these, between 4.6 and 13.9 million clean reads were detected across treatments using HISAT2 software. The total alignment rate including unique and multi-mapped reads with the reference genome ranged between 63 and 98% (Additional file 2, Table S1).

Differential expression analysis was carried out using EdgeR (v.3.43.7) (Robinson et al., 2009) in RStudio Desktop (v. 2023.06.0 build 421) or RStudio server hosted in Cornell University and the Texas A&M University high-performance computing portal running R (v. 4.2.1) framework. Differentially regulated genes with ≤ − 1 or ≥ 1 log2-fold difference with a false discovery rate (FDR) of ≤ 0.05 at each time point were further analyzed. Volcano plots were generated using EnhancedVolcano (v. 1.19.0) [34].

## Gene annotation and gene ontology (GO),KEGG and araCyc enrichment analysis

Gene descriptions and gene annotations were retrieved using the Gene Annotation search tool (https://www.arabidopsis.org/tools/bulk/go/index.jsp; last accessed on 04.06.2023) on curated gene descriptions and annotations from TAIR [35]. GO enrichment analysis was conducted using ShinyGO v0.76 with the following settings: FDR cutoff 0.05, a minimum pathway size 2, and pathway database GO: Biological Process. Visualization of GO enrichment plots were carried out using the built-in tool available in the ShinyGO [36]. DEGs were queried against the KEGG (Release 86.1) and AraCyc (Plant Metabolic Network Release 15.5) databases built into the ShinyGO V0.80* (http://bioinformatics.sdstate.edu/go80/) database. Pathways with a significant FDR enrichment (p ≤ 0.05) were selected for further analysis [36].

Assignments of gene families were carried out using a locally executed uniport database. Amino acid sequences of the genes were extracted using TBTools (v.1_098722) and basic local alignment search tool (BLAST®) of UniProt (release-2022_01) database was generated using BLAST® + executables (v.2.3.10 +) [37]. A BLASTp search of differentially expressed genes (DEGs) was carried out against the UniProt database and based on the resulting family names, were assigned using the UniProt ID mapping tool (https://www.uniprot.org/id-mapping).

## Abscisic Acid (ABA) levels

A separate experiment was set up with Arabidopsis plants with and without TuMV and aphids exactly as described above and up to six samples were collected. Tissue was collected, lyophilized, and ground to a fine powder, and 50 mg was weighed out for each sample. ABA was extracted from tissue using an iso-propanol:H_2_O: hydrochloric acid extraction buffer (2:1:0.005) spiked with 1000 ng/µL of deuterated standard of ABA (( +)-ABA-d6; Cayman Chemical, MI, USA). Extracts were analyzed using a UHPLC system (Thermo Scientific Dionex, Whatham, MA, USA) with a Kinetix C18 column of particle size 1.7 µm, length 150 X 2.1 mm, 100 Å (Phenomenex, USA), and an Orbitrap™-Q Exactive™ mass spectrometer (Thermo Scientific, Whatham, MA, USA). ABA and the standard were separated, identified, and the peak area determined as in [38]. ABA concentrations were quantified by comparing the peak area of the endogenous ABA with 1000 ng of spiked deuterated ABA-d6 and standardized to sample dry mass.

## *Ab initio *promoter analysis

The 1000 bps upstream promoter regions of the differently expressed genes were extracted using the Arabidopsis thaliana Genome Annotation (Starting Version: Araport11; Update date: September 2022) hosted in the TAIR database (https://www.arabidopsis.org/index.jsp). A total of non-redundant 741 promoter cis-elements belonging to taxa group plantae was retrieved from JASPAR database ver. JASPAR_2022_9_NR. Promoter enrichment was carried out using Ciiider software and the site count and coverage *p* values of 0.05 [39]. The frequencies of cis-element binding sites were visualized using the heatmap tool of TBTools (ver.2_008). Identification of ABA-related cis-elements were carried out manually by conducting a comprehensive literature analysis.

## Transcript isoform analysis

A Kallisto (v.0.46.2) transcriptome index was created using the working model transcripts that include both high-confidence and working gene models of the Arabidopsis Col-0 genome assembly (TAIR10). Transcript abundance and estimates were also calculated using Kallisto (v.0.46.2) [40]. Transcript isoform analysis was carried out using IsoformSwitchAnalyzeR (v.2.1.2) [41](Additional file 1). Transcript expression values were imported from Kallisto into IsoformSwitchAnalyzeR using the importRdata function [42]. The isoform switch test was carried out using DEXSeq implemented in IsoformSwitchAnalyzeR [43]. Then, predictions of premature termination codons (PTC) and thereby nonsense mediated decay (NMD)-sensitivity were carried out [43]. The coding potentials of the transcripts were analyzed using CPC2 [44]. Domain architectures of the resulting proteins were identified using the Pfam database [45]. The presence of the signal peptides was inquired using SignalP (v.5.0) [46] and protein disorder was assessed using IUPred2A [47]. Predictions of the consequences of isoforms were conducted and visualized using IsoformSwitchAnalyzeR (v.2.1.2) [43].

## Statistical analysis

For ABA analysis data were log-transformed to meet assumptions of normality and a one-way ANOVA and Tukey analysis was performed to determine differences in factors across treatments. The statistical analyses were performed using R Studio [48].

## Results

### TuMV and aphids regulate greater numbers of transcripts together than alone

**Fig. 1:**
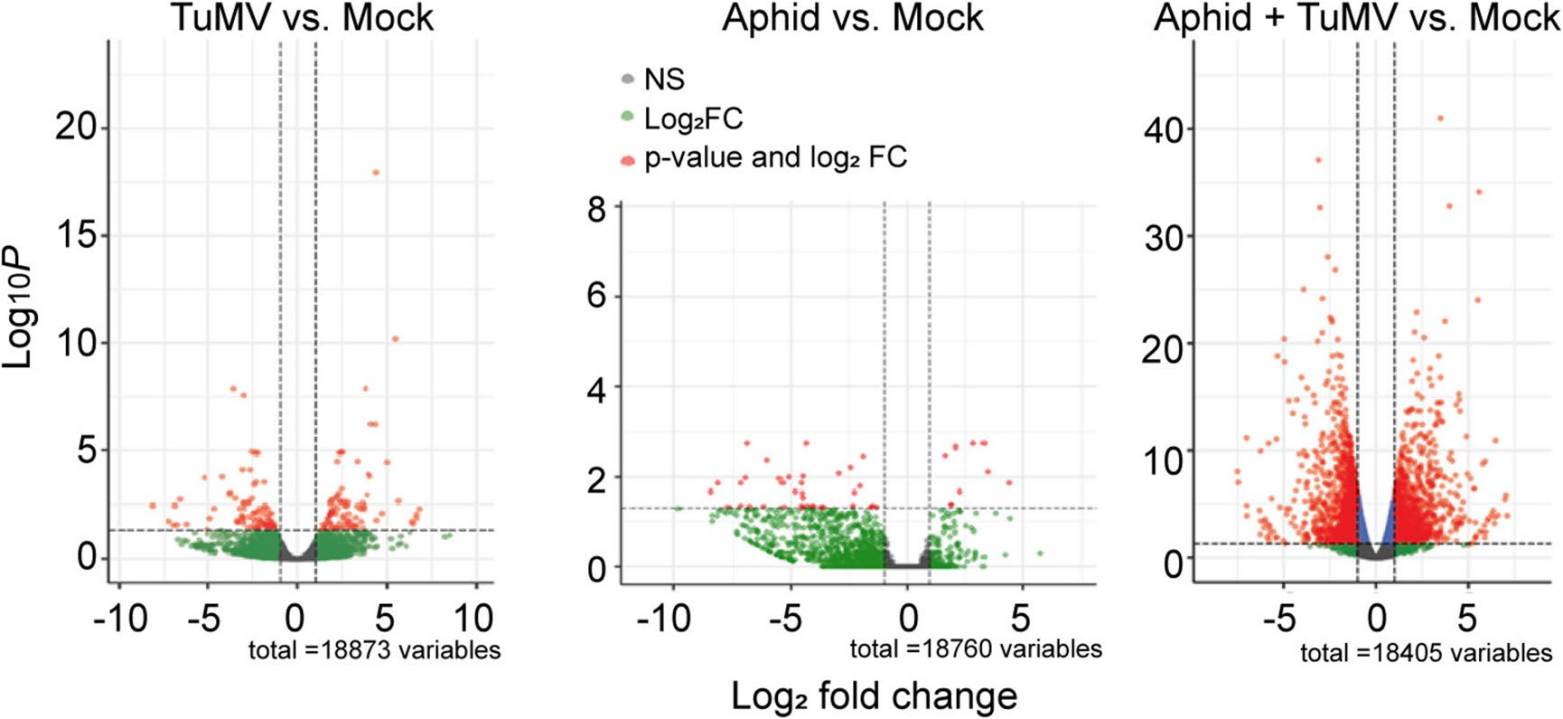
Differentially expressed number of genes (DEGs). Volcano plots show the significantly upregulated genes in blue and downregulated genes in red (*p* < 0.05) when comparing Arabidopsis plants infected with TuMV and healthy (mock) plants, plants infested with aphids and mock plants, and aphids feeding on TuMV infected plants compared with mock plants

We reported Arabidopsis transcriptome responses to *Myzus persicae* aphid-infestation and TuMV infection to investigate the impacts of the combined challenges on plant protein turnover pathways [6]. In revisiting these data, we remapped the raw reads to the reference genome using HISAT2 software and expanded our efforts to obtain a comprehensive view of the plant transcriptomic responses to the individual and combined treatment of aphid feeding and TuMV infection (see Additional file 1 for the experimental plan). Consistent with our previous analysis of differentially expressed genes (DEGs [6]; the log_2_-fold changes were higher in the positive and negative direction when aphids were feeding on TuMV-infected plants than on healthy (Mock) plants treated with aphids or in TuMV infected plants not treated with aphids (Fig. 1). The combination of Aphids + TuMV produced 847 DEGs (339 up; 508 down; Table 1) while aphid-infested plants produced 45 DEGs (9 down, 36 up, Table 1) and TuMV-infected plants generated 177 DEGs (87 up; 90 down, Table 1). A total of 2419 DEGs (1135 up, 1284 down) were exclusive to the Aphid + TuMV treatment and not identified in mock treated plants (Table 1).

**Table 1:** Numbers of differentially expressed Arabidopsis genes during aphid feeding, TuMV-infection, and the combination of both treatments

Comparison of Treatments	Total No. Regulated Genes	Total Upregulated Genes	Total Downregulated Genes
Aphid vs. Mock	45	9	36
TuMV vs. Mock	177	87	90
Aphid + TuMV vs. Mock	847	339	508
Aphid + TuMV vs. Aphid (Novel Aphid with TuMV)	2419	1135	1284
Aphid + TuMV vs. TuMV (Novel TuMV with Aphid)	50	25	25

**Fig. 2:**
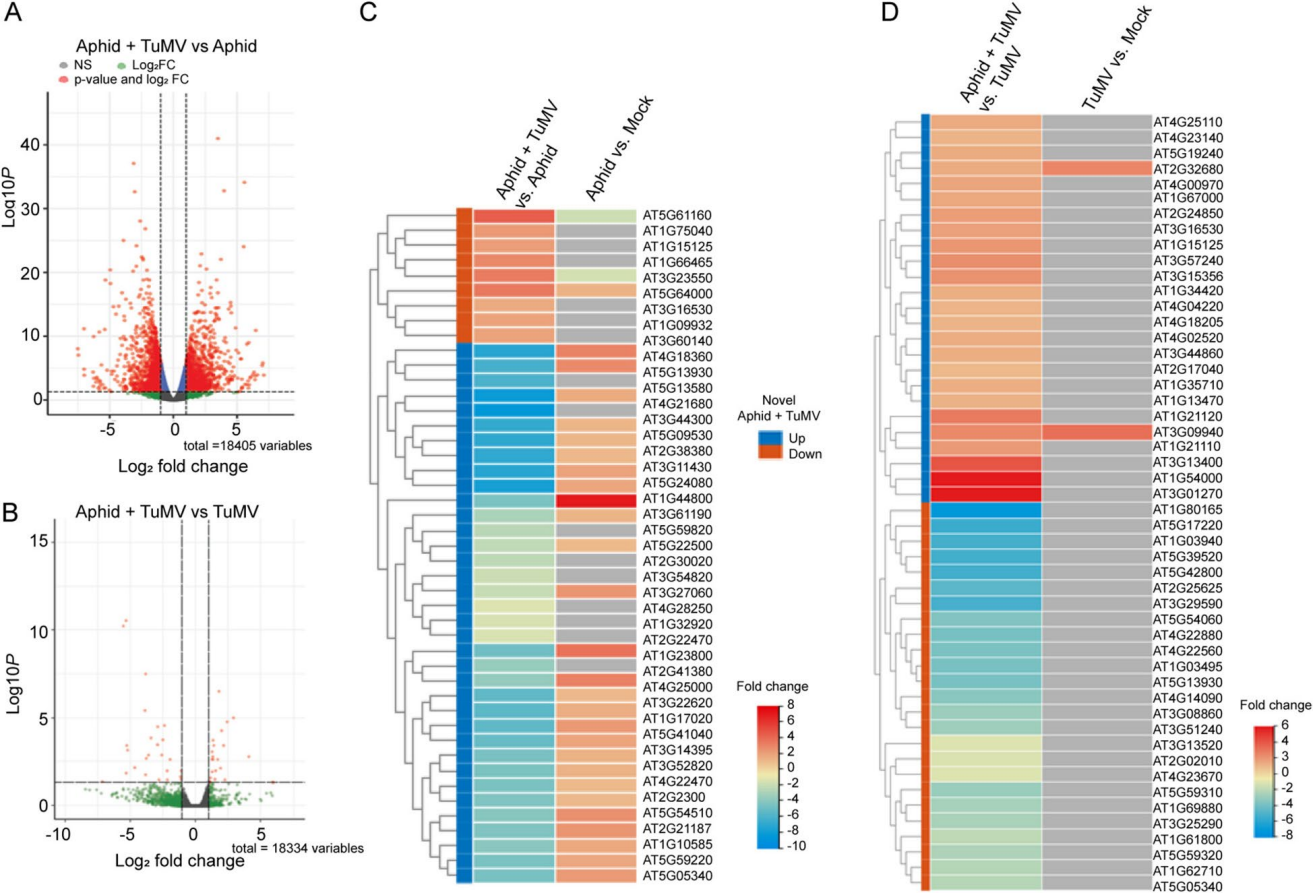
Aphids feeding on healthy versus TuMV infected plants produce different gene expression patterns. **A**, **B**. Volcano plots show numbers of DEGs following treatment of Arabidopsis plants with aphids and TuMV versus aphids alone or TuMV alone. **C**, **D**. Heatmaps showing the relative expression of candidate target genes, that were responsive to the combination of Aphids + TuMV treatment and oppositely influenced by aphid (*n* = 45) or TuMV (*n* = 50) treatment alone. Rows represent genes and columns represent treatments. Clusters of genes were labeled depending upon their unique expression patterns

The coordinated genetic responses to aphids feeding on TuMV infected plants did not appear to be additive based on the numbers DEGs obtained following the individual aphid or TuMV challenge treatments. The log_2_-fold changes were also higher in the positive and negative direction when comparing Aphid + TuMV responsive genes to aphids alone or TuMV alone (Fig. 2A and 2B). A subset of 45 DEGs that were responsive to aphid treatment were oppositely influenced by the combination of Aphids + TuMV treatment (Fig. 2C). Another set of 50 DEGs induced by TuMV treatment showed a different pattern of dysregulation by the combination of Aphids + TuMV treatment (Fig. 2D). Such complex effects on gene expression points to compounding molecular interactions [26].

### Aphids feeding on TuMV infected plants intensify the pattern of stress responsive genes compared to the individual challengers

Virus can stimulate numerous physiological and defense responses through the induction of salicylic acid (SA), jasmonic acid (JA), and gene silencing machinery. Therefore we hypothesize that TuMV infection also primes adaptive machineries for insect compatible interactions by pre-inducing genes. For hypothesis development, we first performed Gene Ontology (GO) enrichment analysis to identify the biological and molecular processes over-represented among the DEG datasets presented in Tables S2 through S9 (Additional file 2). The highest priority was to group genes associated with external and endogenous stimuli, abiotic or biotic stimuli. Since most genes had multiple GO terms, we reported additional GO terms for “response to hormone” or “response to abiotic stimuli” in adjacent columns.

**Fig. 3:**
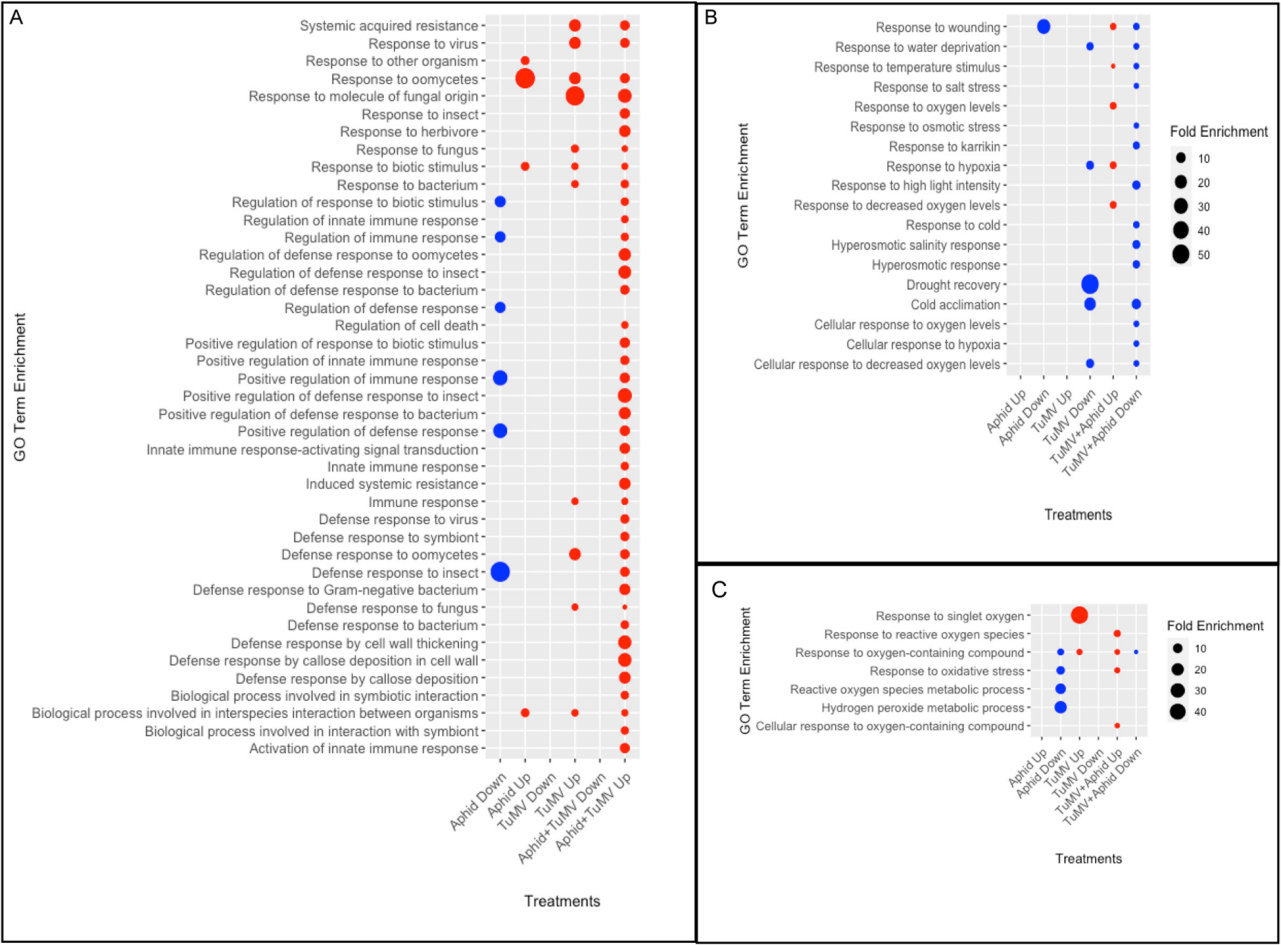
Dot plots from gene ontology analysis representing the fold enrichment of DEGs associated with environmental responses. Rows represent GO terms and columns indicate treatments with aphids, TuMV, or aphids + TuMV. Blue dots represent downregulated and red dots represent upregulated genes. **A**. Analysis of enriched GO terms relating to plant–microbe interactions. **B**. Enriched GO terms relating to abiotic stress. **C**. Enriched GO terms relating to oxidative stress

Dot plots were used to visually compare 42 enriched GO terms relating to host defenses, regulation of defenses, responses to various pathogens, insects, and symbionts across treatments (Fig. 3). Forty GO terms were among the upregulated gene sets following Aphid + TuMV challenge while only 4 or 11 of these GO terms were identified among the upregulated gene sets following treatment with only aphids or TuMV, respectively (Fig. 3A), indicating that the combined challenges amplified the array of cellular defense genes that were activated. A subset of 6 terms that were among the downregulated in response to aphid challenge (Aphid vs. Mock) as well as the upregulated genes when aphids challenged virus infected plants (Fig. 3A) suggesting that there is fine-tuning of gene expression based on the individual or combined challenges. Such fine tuning of gene expression often occurs at the promoter or transcriptional level.

Virus-plant, and aphid-plant interactions, whether compatible or incompatible, are linked to changes in abiotic stress tolerance as well as rapidly changing redox homeostasis [49]. Tables S2 through S5 (Additional file 2) clearly show an abundance of DEGs that are directly or indirectly associated with oxygen metabolism. Many potyviruses, including TuMV, were reported to enhance the activity of antioxidant enzymes, lipid peroxidation, protein oxidation, H_2_O_2_ accumulation, and the loss of chlorophyll levels [50, 51]. Dot plots were used to examine 25 enriched GO terms associated with abiotic stress and oxidative stress or metabolism (Fig. 3B and C). Following TuMV + Aphid treatment, 9 GO biological process terms were upregulated, and 17 GO terms were downregulated (Fig. 3B and C). Treatment with aphids alone or TuMV alone failed to produce any upregulated GO terms in this category once again suggesting that the combination of challengers produced unique genetic responses. Aphid-only treatment and TuMV-only treatment each showed 5 enriched GO terms were downregulated and were categorically different from each other (Fig. 3B and C). As before, the genetic responses to aphids’ feeding on virus-infected plants were the opposite of aphids feeding on healthy plants [6].

### Regulatory changes associated with aphids and TuMV infection point to key metabolic and hormone regulated molecular networks

The expanded list of DEGs in the Aphid + TuMV treatment compared to aphids alone or TuMV alone includes genes encoding catalytic and biosynthetic enzymes across the plant metabolic network. Enzymes are up and downregulated to activate or deactivate branch metabolic pathways to support adaptive responses to environmental challenges [52]. Metabolic pathways controlling polyphenol and phytohormone synthesis are often controlled by positive and negative feedback loops between the metabolites and their enzymatic genes [53, 53–55]. Therefore, GO analysis of DEGs was performed to identify key secondary metabolites and phytohormones that influence cellular adaptive responses.

**Fig. 4:**
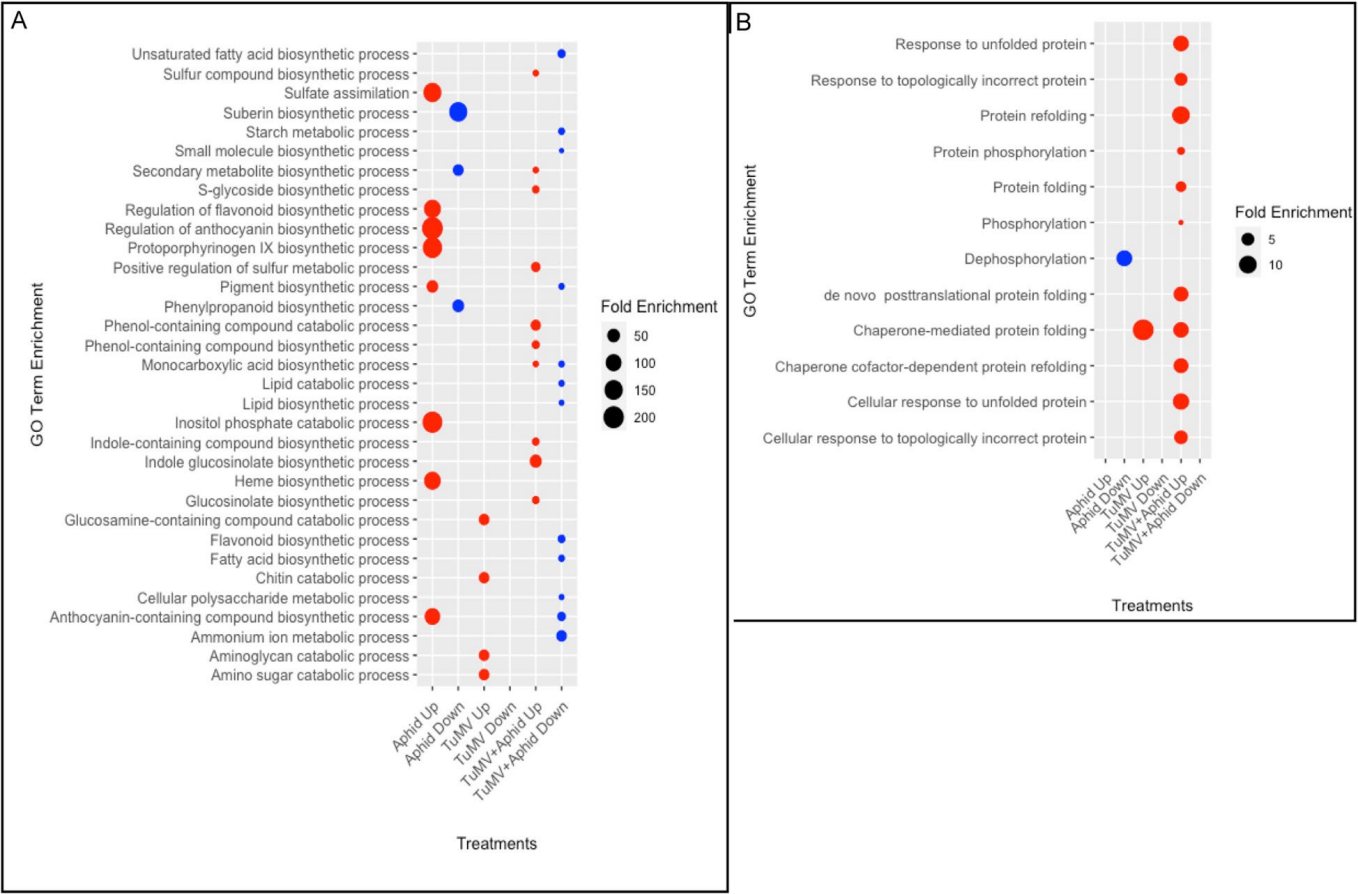
Dot plots from gene ontology analysis representing the fold enrichment of DEGs. **A**. Rows represent biosynthetic and metabolic pathways. **B**. Rows represent GO terms for protein maturation processes

Thirty-three GO terms for secondary metabolic and biosynthetic processes were examined revealing various GSLs (glucosinolates), phenylpropanoids, flavonoids, fatty acid biosynthesis, and anthocyanin pathway genes and regulators **(**Fig. 4A and Additional file 2**,** Tables S2 through S9)**.** The profound and unique patterns of DEGs are attributed to various GO terms in plants treated by aphids alone or TuMV alone and were not additive when the combined challengers were applied to plants. Since GO terms are organism independent and provide general categorization of genes into biological or molecular pathways, we employed KEGG and ARACyc databases to gain more precise insight into the changes in gene expression associated with key Arabidopsis metabolic pathways (Additional file 2, Table S10). More genes engaged in GSL biosynthesis from tryptophan, indole-GSL activation, indole-3-acetate inactivation were upregulated by TuMV + Aphids compared to individual treatments. For TuMV + Aphids compared to TuMV alone there are 3 GSL biosynthetic genes. For TuMV + Aphids compared to aphids alone there were 7 GSL biosynthetic genes, 5 indole-GSL activation genes, 4 indole-3-acetate inactivation IV genes (Additional file 2, Table S10). This observation coincides with the upregulated GO terms including the biosynthesis and catabolism of phenol containing compounds, sulphur compounds, GSLs, and indole containing compounds (Fig. 4A). Given that indolic-GSLs are sulfur and nitrogen containing compounds involved in plant defenses against herbivores and pathogens, associated with auxins and phytoalexins, these GO enrichment data in Fig. 4 combined with data from Figs. 2, 3, and Table S10 support the hypothesis that a combination of plant defenses are stimulated by these dual challengers [56]. Importantly, since the differential expression of GSL and indolic-GSL biosynthetic genes are regulated by transcription factors and plant hormones including abscisic acid (ABA), jasmonic acid (JA), salicylic acid (SA), and ethylene (ET) these data suggest a significant role for plant hormone biosynthesis in responding to the combined challengers [56]. KEGG analysis identified 25 genes and AraCyc identified 11 genes in phenylpropanoid and ubiquinone biosynthesis are largely upregulated by the combined challengers.

More genes encoding enzymes controlling the biosynthesis or modifications of flavonoids, (pro)anthocynanidins, leucocynanidins are downregulated for Aphid + TuMV challenges than the individual challengers (Additional file 2, Table S10). These outcomes could be tied to plant phytohormones such as auxin, JA, and/or GA which monitor transcriptional controls of the expression and repression of flavonoid biosynthetic genes. Moreover, recent reports indicate that TuMV specifically downregulates genes contributing to flavonoid and anthocyanin biosynthesis [57]. Anthocyanins are important antioxidants and contribute to TuMV resistance [60].

TuMV infection is well known for altering transcriptional programing through interactions with the cellular unfolded protein response machinery [13, 14, 58] however little is known about how aphids influence cellular reprogramming at the same level **(**Fig. 4B). Heat maps were generated using 12 GO terms surrounding protein folding and maturation. As expected TuMV infected plants showed a tenfold enrichment of genes involved in chaperone-mediated protein folding [13, 14, 58–60] and no terms were among the downregulated genes (Fig. 4B). Aphids feeding on TuMV infected plants led to significant enrichment of 11 GO terms and none were downregulated. KEGG analysis identified 21 genes involved in protein processing in the endoplasmic reticulum (ER) and 10 genes involved in ribosome biogenesis that are uniquely influenced by TuMV + aphid challenges rather than the individual challengers alone (Additional file 2, Table S10). Looking carefully at the KEGG list of genes involved in protein processing in the ER, the majority of factors are chaperones or co-chaperones. Regarding ribosome biogenesis, these genes include pre-RNA processing and transcription factors. These data suggest that nuclear and nucleolar reprogramming of the protein maturation machinery takes precedence over the activation of the canonical UPR signal transduction pathways for maintaining protein maturation and quality control in response to these combined challenges [13, 61].

Chloroplasts are vital for redox regulation, communicate with the nucleus through retrograde signaling and are active in biosynthesis of compounds and phytohormones involved in stress responses [49]. KEGG and AraCyc analysis captured evidence that photosynthesis is downregulated while chlorophyll degradation is upregulated. Photorespiration and the enzymes in the calvin cycle are downregulated by the combined challengers more than the individual treatments (Additional file 2, Table S10). Chlorophyllase, which is a chlorophyll-degrading enzyme, is upregulated in the TuMV alone as well as TuMV + Aphid datasets. Several ferredoxin thioredoxin family proteins are among the TuMV + Aphid datasets (Additional file 2). Among the 18 upregulated genes are enzymes involved in the integrity of photosynthetic processes and membranes and starch mobilization. Most of the downregulated genes are involved in redox regulation, as well as light harvesting components of photosystem I and II. Chloroplast generated reactive oxygen species (ROS) can function as a retrograde signal that can modify the nuclear gene expression in response to environmental stress. Interestingly there were three downregulated genes (none induced) following treatment with TuMV alone, contrasted by 18 upregulated and 120 downregulated genes in the TuMV + Aphid dataset indicating that when aphids feed on virus infected plants, they have a greater influence on redox stress responses.

The combined enrichment of GO terms, KEGG, and AraCyc analysis clearly point to the significant reprogramming of gene expression which are typically driven by transcriptional factors and co-regulators. In addition, evidence that pre-RNA processing affecting ribosome maturation led us to hypothesize that alternative RNA splicing may represent another level of defense against these combined challengers. The following analysis examines both levels of gene regulation.

### TuMV and aphids combined influence ABA-dependent host transcriptional responses to aphid infestation

**Fig. 5:**
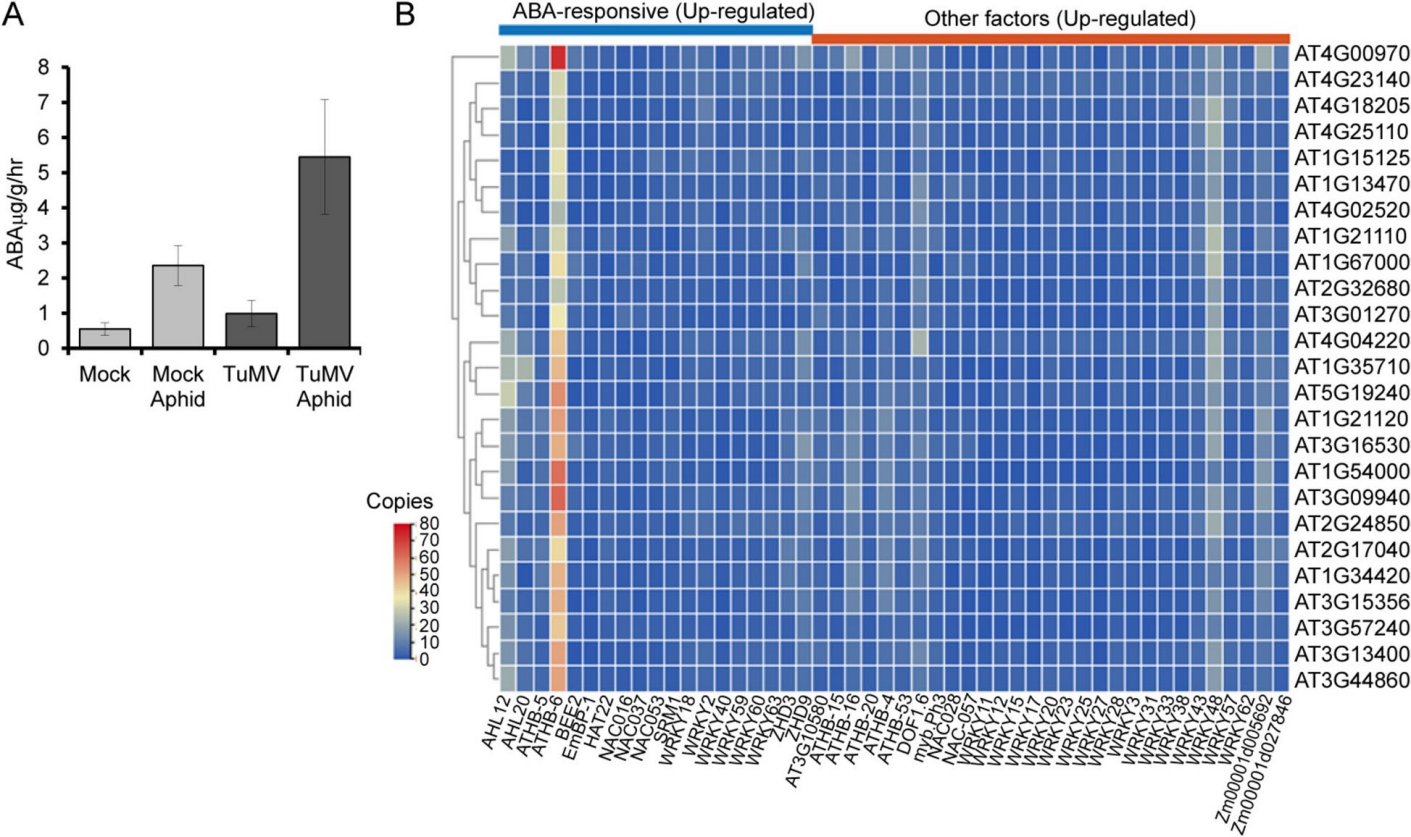
ABA levels and genes with ABA responsive elements that are DEGs in this study. **A**. Graph shows the concentration of ABA in mock, aphid treatment, TuMV infection, and aphids feeding on TuMV infected plants. A one-way ANOVA was conducted, followed by a Tukey post hoc test to determine differences between treatments (*P* < 0.05). **B**. Genes with ABA responsive cis regulatory elements (CREs) in the promoters. The number of each category of ABA response and other CREs are identified in each column

The role of SA and JA signaling in regulating virus defenses has received more attention than the role of ABA. There are a few examples where ABA functions to mediate plant-virus interactions partially through effects in the RNA silencing pathway [62–65]. TuMV is reported to alter ABA homeostasis, differentially influence the expression of ABA-responsive genes, and increase host sensitivity to water stress [66]. Here, ABA concentrations were elevated 2.5-fold in aphid infested Arabidopsis plants and fivefold elevated when aphids were feeding on TuMV infected plants (*P* < 0.05; Fig. 5A). When manually curating genes in the Supplementary Tables (Additional File 2), there were wider number of genes that were identified in the Thalemine and TAIR databases to be engaged in hormone responses, especially ABA, than determined via GO analysis.

To understand the influence of ABA on transcriptional reprogramming in response to aphids and TuMV infection, we selected 1,000 bp upstream of the predicted transcription start site for the 25 genes that were upregulated when comparing Aphids + TuMV to TuMV infected plants (Table 1) and the 1135 genes upregulated by Aphids + TuMV when compared to aphids alone (Suppl. Fig. S2, Fig. 5B). The 25 genes that are uniquely responsive to Aphids + TuMV include receptor-like proteins, protein kinases, and other enzymes linked to biotic and abiotic stress. Multiple types of TFs are involved in ABA responses including the ABA-insensitive (ABI) factors, Group A basic region/leucine zipper (bZIP) factors which bind to the ABA responsive (AREB/ABF), cis regulatory elements (CREs) in promoters NAC domain factors, WRKY factors, MYB factors, C2H2-type zinc finger (ZFPs), basic helix-loop-helix (bHLH) factors [67–73]. Therefore, we examined a wide range of potential TF CREs to best discover crucial ABA-related factors. Our analysis produced 48 TF CREs linked to hormone signaling. The most striking outcome was that the ABA-response ATHB-6 binding site was represented between 20 and 80 copies per gene. The WRKY48 binding site, which is not ABA-responsive per se, was the second highest representation, between 10 and 25 copies (Fig. 5B). Interestingly, WRKY48 was initially reported to be a transcriptional activator that represses plant basal defenses including PR gene expression leading to enhanced growth of *Pseudomonas syringae* (DC3000) [74]. While ATHB and WRKY factors represent families of regulators involved in biotic and abiotic stress and ABA signaling [70], this is the first report suggesting ATHB-6 as well as WRKY48 may be essential for interactions involving host tolerance to aphids and plant virus infection. The binding sites for two additional ABA responsive factors, AHL12 and DOF1.6, occurred an average of ≥ 10 copies per gene (Fig. 5B). Further studies will be needed to determine if these factors interact in a meaningful way to influence gene expression.

### ABA independent changes influencing aphid and TuMV related plant drought tolerance

Given that ABA is a primary phytohormone that regulates plant responses to drought, we expect that the heightened ABA levels could partially explain the improved drought tolerance seen in TuMV infected plants. GO analysis presented in Fig. 3 shows clusters of genes associated with the term “response to water deprivation” as downregulated by aphid + TuMV treatment, although genes associated with the GO term “drought recovery” seem unaffected. The Arabidopsis *9-*cis*-epoxycarotenoid dioxygenase-3* (*AtNCED3*) is required for ABA biosynthesis and its transcripts, along with *ENOLASE 2* (*ENO2*) were shown to be drastically induced in drought-treated plants [75]. *AtNCED-3* and other ABA biosynthetic genes influence stomatal closure [76]. DREB/CBF proteins are AP2 transcription factors that also influence *MEDIATOR 16/SENSITIVE TO FREEZING 6* (*MED16/SFR6*; AT4G04920; upregulated in Additional file 2, Table S6) expression [77]. *RESPONSIVE TO DESICCATION 29A* (*RDR29A*) expression is controlled by MED16/SFR6 and linked to drought stress.

Table S7 (Additional file 2) lists genes that are downregulated in response to aphids feeding on TuMV infected plants and includes *AtNCED3* (AT3G14440) and seven other genes linked to the regulation of stomata closure: *AT2G40820, AT3G10660, AT5G37500, AT2G04570, AT4G17970, AT3G01500, AT5G65590*. The SNF1-related protein kinases 2 (SnRK2) are also ABA inducible and directly phosphorylate the ABA-responsive element-Binding Factor (ABF)-type transcript factors [72, 78]. In Table S3 and S7 the *SnRK2.5* (AT5G63650) are downregulated by TuMV infection and Aphids + TuMV treatment. These data suggest that the ABA dependent transcriptional regulation of these factors may be suppressed rather than induced. Interestingly, fibrillarin (At4G25630), which is a nucleolar protein that interacts with viral proteins in the nucleus or nucleolus to disable anti-viral responses and promote infection. For potyviruses, the VPg interacts with fibrillarin in the nucleus and researchers have speculated that the interaction affects host transcription and pre-mRNA processing for host-gene shut off [79].

The Tables S3, S6, and S7 identify *DREB1A, DREB2C, DREB4, DREB26, RAP2.4,* and *TG* (At4G25480, AT1G21910, AT1G78080, AT2G40340, AT1G36060, AT5G52020) among the differentially regulated genes suggesting fine tuning among these transcription factors to regulate the physiological conditions of challenged plants.

### Regulation of transcript isoform accumulation

ABA is also known to induce RNA alternative splicing (AS) in response to environmental challenges and growth [80]. New high throughput sequencing approaches have revealed that plant and animal infecting viruses modulate RNA alternative splicing (AS). There are few examples describing AS as a mechanism influencing immune responses in virus-host interactions [81–83]. In the case of sugarcane mosaic virus (SCMV) the AS maize phytoene synthase 1 (*ZmPSY1*) ensures persistent virus infection while decreasing chloroplast damage [20]. Others suggest that viruses may stimulate AS to highjack host factors from the normal cellular functions to aid in virus replication and accumulation. For example, proteins with intrinsically disordered regions (IDRs) involved in cell signalling, often serve as hub proteins for multiple partners involved in different mechanisms and virus influences on mRNA splicing could favor pro-viral mechanisms [84]. In mammalian systems, viruses such as influenza or are also known to alter the expression of the nucleolar ribonucleoprotein (RNP) complex that removes introns from pre-mRNA as a means to disrupt host gene expression, favoring viral gene expression [20, 85]. Such viral interventions on pre-mRNA splicing can favor host transcripts that are targets for nonsense mediated decay.

Table 2 shows that there are 21 and 30 genes with differentially used isoforms as the result of aphid infestation alone or TuMV infection alone, respectively. The combination of Aphid + TuMV saw an increase of number of genes to 36. When we compare these datasets there are 22 novel genes resulting from Aphid + TuMV compared to aphid alone and 20 genes when compared to TuMV alone. Only 30–50% of genes with isoform switches encoded proteins with IDRs and between 30 and 50% of these showed isform switches where the IDRs were unequally represented (Table 2). Given the hypothesis that AS can expand the complexity of the cellular proteome during biotic stress, very few genes show alternate isoform usage in comparison to the overall number of DEGs. However, these data provide the first evidence that aphids along with TuMV can influence pre-mRNA splicing carried out by the spliceosome. There were 29 isoform switches that were exclusively influenced by Aphids + TuMV when compared to aphids alone and 21 exclusive isoform switches when compared to TuMV alone (Table 2). Interestingly RNA-dependent RNA polymerase 6 (RdR6) which is critical in gene silencing defenses to virus infection, was among the genes with isoform changes. There were many other genes involved in pathogen defense or abiotic stress. There were many TFs or mRNA binding factors affecting RNA metabolism including translation and pre-mRNA splicing. Several factors were associated with chloroplast metabolism (Table S11 and S12). Interestingly the U2AF65A (AT4G36690) which is an important subunit for U2 small nuclear ribonucleoproteins (snRNP) was identified in Supplementary Table 12. ABA is also known to regulate *U2AF65A* splicing which in turn influences ABA-mediated flowering and drought stress. While protein functional domains there were only 2 factors, based on GO terms, for which alternate isoforms resulted in a change in protein subcellular targeting. At3G2360 is an mRNA binding factor for which one isoform associates with the Golgi and vacuole, and the other isoform is extracellular. The other factor is AT1G23750 which is involved in nucleic acid binding and chlorophyll biosynthesis and one isoform is nuclear while the alternative isoform is cytoplasmic (Table S11 and 12). One explanation is that virus infection or aphids might highjack these factors for compatible interactions although further investigations are needed to study these interactions.

**Table 2:** Assessment of genes with different isoforms

Comparison of Treatments	nr Isoforms	Nr Switches	nr Genes	nr Genes with IDRs	Transcripts with unequal IDRs
Aphid vs. Mock	34	24	21	8	3
TuMV vs. Mock	51	33	30	9	3
Aphid + TuMV vs. Mock	57	41	36	17	6
Aphid + TuMV vs. Aphid (Novel Aphid with TuMV)	35	29	22	10	6
Aphid + TuMV vs. TuMV (Novel TuMV with Aphid)	29	21	20	10	3

Footnote: nrIsoforms are number of differentially used isoforms, nrSwitches: one isoform is dfferentially used and another isoform with opposite change, nr genes are number of genes with at least one differentially used isoforms. nr Genes with IDRs: number of genes with at least one differentially used isoform that encodes a protein with an intrinsically disordered region (IDR)

**Fig. 6:**
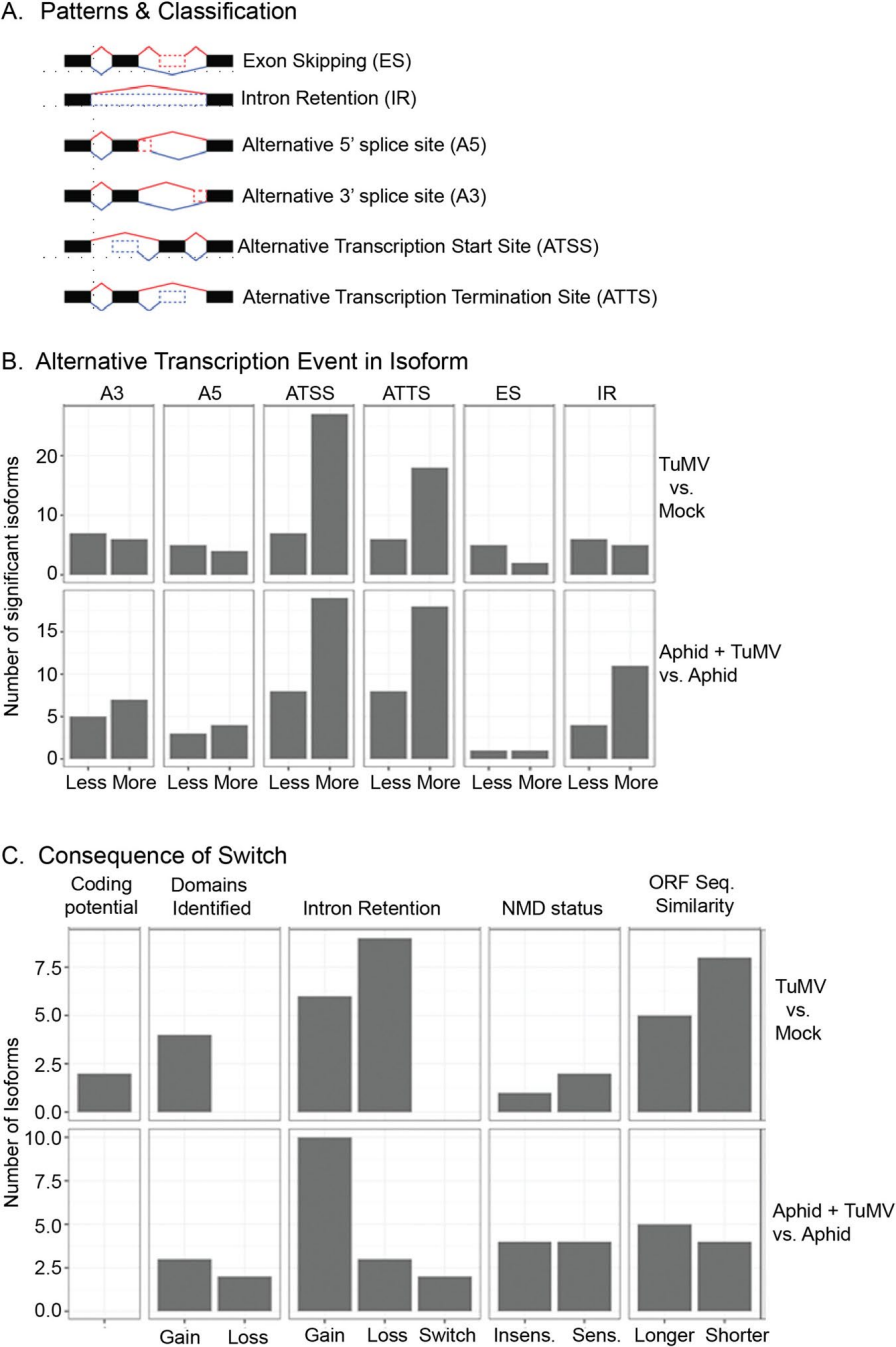
Isoform enrichment analysis. **A**. Illustration of classes of isoform changes that can occur during transcription and pre-mRNA processing. **B**. Number of significant isoforms according to the alternative transcription event seen when comparing TuMV infected and mock plants, or aphids + TuMV and aphid infested plants. **C**. Consequence of the alternative isoforms based on the categories presented across the top of each grid. The Y axis shows number of isoforms influenced and the X axis indicates the gain or loss according to the category

The dominant mode for alternative transcription events in TuMV infected plants and aphids + TUMV infected plants was the production of isoforms via alternative transcription start (ATSS) and termination (ATTS) sites. There was also a positive shift in the number of intron retention (IR) events when we compared Aphids + TuMV to aphids alone (Fig. 6A, B). More often intron retention resulted in a gain in coding sequences. For the most part, aberrant RNAs are degraded through nonsense mediated decay (NMD). NMD sensitive isoforms were slightly increased by TuMV infection, but not altered by aphids feeding on TuMV infected plants when compared to Aphids-only treatment (Fig. 6C). There was little evidence linking ABA signaling to AS pathways in aphid and TuMV response based on the identified genes or the pattern of splicing events which commonly include intron retention, and alternative 5’ or 3’ splicing sites.

**Fig. 7:**
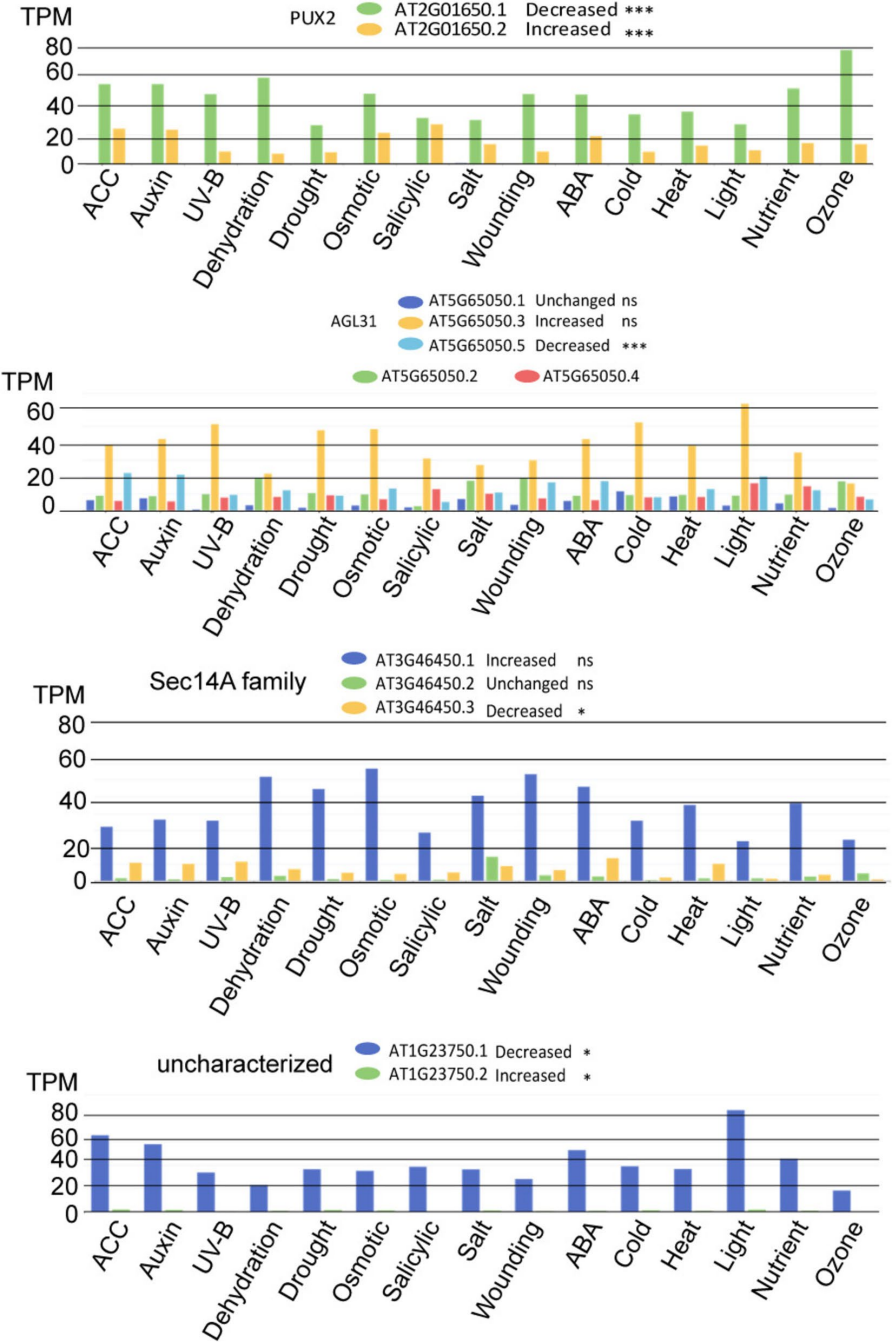
Isoform enrichment of four genes. Colored bars represent isoforms indicated in the legends for each graph. X axis indicates the environmental or hormonal challenge affecting isoform accumulation. Y axis indicates transcripts per million

To test the hypothesis that AS of transcripts is a feature of plant adaptation to environmental stress, we examined the AS events of four genes that were found to be uniquely regulated by Aphids + TuMV when compared to TuMV or aphids from Tables S11 and S12: PUX2, AGL31, Sec14-family protein, and an unknown factor in relation to a wide range of environmental stressors as reported in public databases (Fig. 7). Interestingly, the levels of PUX2 isoform 1 decreases while isoform 2 increases in the presence of Aphid + TuMV when compared to aphids alone suggesting that TuMV infection preconditioned the cells for further abilities to respond to aphid infestation. Across 15 treatments involving abiotic stress or hormones, only SA treatment showed a rise in isoform 2 and decrease in isoform 1. All other treatments, including ABA retained higher isoform 1 than 2 levels (Fig. 7). For AGL31 isoform 1 and 3 are not statistically significant while isoform 5 is downregulated by the combined aphid + TuMV treatment. Across the same 15 treatments in Fig. 7, the isoforms 3 and 5 are widely altered due to environment or hormone challenges. Sec14A isoforms 1 increases while isoform 3 decreases in the Aphid + TuMV dataset, and across the 15 treatments. In the case of the uncharacterized AT1G23750, isoform 1 is decreased and isoform 2 is increased. The opposite is true for abiotic stress treatment (Fig. 7). These data suggest that complex gene regulatory networks affecting the accumulation of transcript isomers underly Aphid + TuMV infection overlap genetic responses to other environmental challenges. These data point to fine tuning of gene expression involving pre-mRNA processing alongside differential gene expression.

## Discussion

This transcriptome study was undertaken to understand the landscape of cellular reprogramming that occurs when aphids are feeding on TuMV-infected or healthy plants. Remarkably fewer genes were influenced by aphids alone and TuMV alone compared to the combined challengers. It is arguable that since the plants are already virus-infected that genetic modifications by the combined challengers are important to create an environment that favors acquisition feeding, enables vector-borne transmission, or promotes aphid reproduction. Regarding plant response to aphids, there has been very little information available concerning the potential signaling hubs that integrate sensing functions relating to the virus and its vector.

The cumulative assessment of genes that are exclusively affected by aphids alone or Aphids + TuMV was examined. Aphids feeding on mock Arabidopsis plants specifically downregulate suberin biosynthetic processes, regulators of defense responses or responses to external stimuli such as wounding or insects, and phenylpropanoid biosynthetic processes. At the same time, there is exclusive upregulation of sulfate assimilates, certain pathogen defense responses, regulation of flavonoid, anthocyanin, and pigment biosynthetic processes, and inositol phosphate dephosphorylation. Genes that are exclusively downregulated by aphids feeding on TuMV infected plants encode factors engaged in cold responses, photosynthetic processes, NADH and NAH(P)H dehydrogenase complex assembly, lipid and fatty acid biosynthesis, and circadian rhythms.

Genes that are exclusively affected by TuMV alone or Aphids + TuMV also presented GO terms that were exclusively influenced by TuMV or the combination of challengers. Among the upregulated were genes involved in systemic acquired resistance, SA metabolism, fungal defenses, immune responses, and leaf senescence. Genes that are exclusively downregulated by Aphids + TuMV that are not influenced by TuMV alone include genes involved in responses to JA, ABA, water deprivation, and UV-B. This was surprising as we found plants infected with TuMV had higher ABA levels (Fig. 5A), and in previous studies we demonstrated the TuMV-infected plants are more resilient to drought [86]. These results suggest that the increase in drought protection does not involve stomata closure or osmotic protectants, but other mechanisms of drought resistance [87].

Around 50% of the DEGs that were altered in response to aphids, TuMV, or the combination of both challenges were related to abiotic and biotic stress responses (Tables S2-S8), plant development, hormone signaling, and innate immunity. Herbivore-associated molecular patterns (HAMPS), pathogen-associated effector triggered immunity (ETI) and pattern triggered immunity (PTI) involve common phytohormone signaling pathways led by salicylic acid (SA) and jasmonic acid (JA). In this study, 29 genes associated with SA and JA-dependent defense pathways were upregulated when aphids were feeding on TuMV infected plants, but not on healthy plants or in plants that were only infected with TuMV. The list includes PAL1 (phenylalanine ammonia lyase 1; At2G37040) and SARD1 (At1G73805) which are essential for SA biosynthesis. A greater number of genes involved in JA metabolism were observed including several lipoxygenases, jasmonate induce oxygenase, and jasmonate-ZIM (JAZ) domain proteins [88, 89]. DEGs associated with oxidative stress and ROS-scavenging include cytochrome P450 monooxygenases (CYPs) and 2-oxoglutarate-dependent oxygenases (2-ODO), superoxide dismutase, catalase, and peroxidases. These families participate in a broad range of cellular metabolic processes and are important for plant-environment interaction [90, 91]. KEGG and AraCyc analysis specifically shows flavonoid and anthocynanin biosynthetic processes are altered by TuMV alone or Aphids + TuMV [60].

Receptor like proteins (RLP) play significant roles in plant immunity, response to environmental challenges, as well as in plant growth and development [92, 93]. Comparing aphids feeding on TuMV infected plants to feeding on healthy plants, the upregulated genes (Table S6) included 13 leucine rich repeat (LRR) proteins, 32 RLPs and receptor like kinases (RLKs), 13 serine/threonine kinases, and 25 other kinases. Among the downregulated gene are 11 LRR proteins, and 44 receptor kinases and other kinases (Suppl Table 7). RLPs are typically located at the cell surface with extracellular LRRs, as well as receptor like kinases (RLKs), and intracellular kinases. For example, RLP38 (At3G23120) which is related to Clavata 2 (CLV2) was upregulated by TuMV and by aphids in the presence of TuMV, but not by aphids alone (Supplementary Table 2 and 6). Other Clavata signaling pathway genes are downregulated by aphids feeding on TuMV infected plants, but not by aphids alone include CLV1 (AT1G75820), CLV3 (AT1G70895), CLE9 (AT1G26600), CLE10 (AT1G69320), CLE17 (AT1G70895), CLE21 (AT5G64800), CLE26, (AT1G69970), CLE42 (AT2G34925), BAM2 (AT3G49670) (Supplementary Table 7). CLAVATA -type receptors are known for their role in apical meristem development [94], and more recently are shown to be crucial in plant response to abiotic stress, plant–microbe interactions, and parasitic nematodes which also can secrete CLE-like effectors into plants [95]. Aphids potentially deliver effector molecules through their saliva into plants, and it is worth speculating that aphids may deposit CLE peptides which could impact host gene expression or feedback loops regulating the Clavata signaling pathway [95]. Not many LRR proteins that recognize aphid signals have been identified and as plant viruses are obligate pathogens it is unclear if virus infection increases or decreases expression of defense related RLPs and RLKs that would otherwise respond to aphid feeding.

ABA is also important for diverse plant-pathogen interactions, abiotic stress, as well as plant growth and development. MED16 is a component of ABA-transcriptional regulation bridging the ABF transcription factors with RNA polymerase II. MED16 plays a crucial role in defense response against aphids and TuMV infection and we recently showed that its nuclear localization if negatively impacted by NIa-Pro [96]. In this study we identified a number of genes that are regulated by ABA and MED16 that are downregulated when aphids infest TuMV infected plants compared to healthy plants. While dysregulation could be explained by disarming MED16 the robust downregulation points to the possibility of an unknown suppressor that competes with MED16 at the same promoters. This data support a model in which MED16 is a potential signaling hub for precise genetic changes in response to TuMV and aphids.

One significant theme that stands out from the data provided in the supplementary tables is that many of the genes that are differentially regulated exemplify crosstalk between cellular immunity, cell survival, adaptation, and responses to environmental stress. These genes include factors that are essential for cell survival but are either hijacked by virus infection or have dual functions in defense or adaptation. For example, when plant potyviruses, including TuMV, initiate infection the first step is translation of the viral replication associated proteins followed by synthesis of antigenomic and progeny RNAs. In leaves that were mechanically inoculated with TuMV or inoculated by viruliferous aphids, genes that were involved in mRNA stability, protein synthesis, protein folding, and maturation were differentially regulated (Tables S2, S3, S4, and S5). This same machinery is important for cellular survival as well as virus infection. Only Pumilio 12 was induced by both TuMV inoculation and viruliferous aphids. TuMV alone induced four chaperones that are typically induced by a wide range of stresses to ensure protein folding while downregulating four other factors that regulate translation and protein turnover (Tables S2 and S3). In plants treated with viruliferous aphids, there are 14 upregulated genes and 17 downregulated genes associated with mRNA and tRNA maturation, stability, and turnover; 43 protein chaperones were upregulated, and 30 chaperones were downregulated; 16 translation and protein modifying enzymes were upregulated while 4 were downregulated; and 30 factors involved in protein turnover and autophagy were upregulated while 25 were downregulated. Clearly viruliferous aphids cause robust changes in gene expression influencing plant mRNA production and protein maturation. This could be explained as evidence of cellular reprogramming to accommodate herbivory and virus infection as well as supporting genetic defense responses.

Ten transcription factors or co-factors were upregulated and six were downregulated. These factors are known to influence gene expression involved in plant growth and defense indicating that TuMV infection directly influences changes in nuclear gene expression (Table S2 and S3). Other metabolic changes associated with nucleotide metabolism and basic enzymatic biosynthetic or degradation functions were altered although a wider array of factors were downregulated. Among the downregulated genes were a variety of factors involved in plant growth, pollen, and reproductive development. Given the often-described genetic trade-offs between growth and immunity that are often linked to hormone metabolism, we examined the downregulated dataset for genes involved in hormone signaling and identified only four genes involved in auxin or ABA metabolism. More genes were downregulated by TuMV infection than were induced and these were categorically different from the upregulated genes.

Stress-induced AS is a critical mechanism for the direct regulation of gene transcription, helping plants to rapidly adapt to environmental challenges. ABA treatment and other abiotic stress treatments of Arabidopsis influence the expression of pre-RNA splicing factors as well as epigenetic modifications controlling intron splicing [80, 97, 98]. Studies show that truncated proteins produced from alternate mRNAs can often interact with the same substrate targets but may recognize different co-factors. Epigenetic factors controlling AS may present as reversible phosphorylation of splicing factors, histone modifications, or DNA methylation affecting the speed of RNA polymerase II elongation [98]. Putative alternative splicing events have been recently reported in studies involving other plant viruses such as bamboo mosaic virus, bean common mosaic virus, and panicum mosaic virus [19, 64, 99].

In Tables S11 and S12, we observed a steep increase in the use of alternative transcription start sites and termination sites as an outcome of aphids feeding on TuMV versus healthy plants. Surprisingly, both aphids and TuMV treatment showed altered isoform ratios for mRNAs encoding transcription factors or factors that can be classified as epigenetic regulators or AS. These include mRNA binding proteins, RNA pol II associated factors, histones or histone modifying enzymes, and topoisomerase I alpha [98]. Most of the genes with shifting isoform usage encoded, regulatory factors in the nucleus, chloroplast, mitochondria, or peroxisomes. Figure 7 point out that database records feature alternate mRNA ratios derived from studies of hormone treatment or abiotic stress. Much less is known about the role of AS in regulating host adaptation or defenses to virus infection or aphid herbivory. While the mechanism for virus or vector induced AS in plants is not known, it is possible that expression of Med factors which regulate RNA pol II, downregulation of fibrillarin which regulates mRNA splicing, and perhaps the recruitment of different splicing factors in the splicing complex could explain this phenomenon.

To summarize, large numbers of genes that are differentially regulated exemplify crosstalk between cellular immunity, cell survival, adaptation, and responses to environmental stress. These include factors that are essential for cell survival and some that are either hijacked by virus infection or have dual functions in defense or adaptation. Alternative splicing of pre-mRNAs serves to add or remove protein domains, and this appears to be an essential component of the arms race between viruses, herbivores, and their hosts.

## Supplementary Information

Supplementary Material 1.

Supplementary Material 2.

Supplementary Material 3.

## Data Availability

The datasets generated during this study are available at the NCBI SRA (PRJNA60524) and 10.3390/v14061341. All other data are available upon request.
