# Supplementary figures and images for: Comprehensive transcriptomic analysis reveals turnip mosaic virus infection and its aphid vector Myzus persicae cause large changes in gene regulatory networks and co-transcription of alternative spliced mRNAs in Arabidopsis thaliana
Source: BMC Plant Biol. 2025 Jan 30;25:128. doi: 10.1186/s12870-024-06014-3 (PMC11780806; doi:10.1186/s12870-024-06014-3)

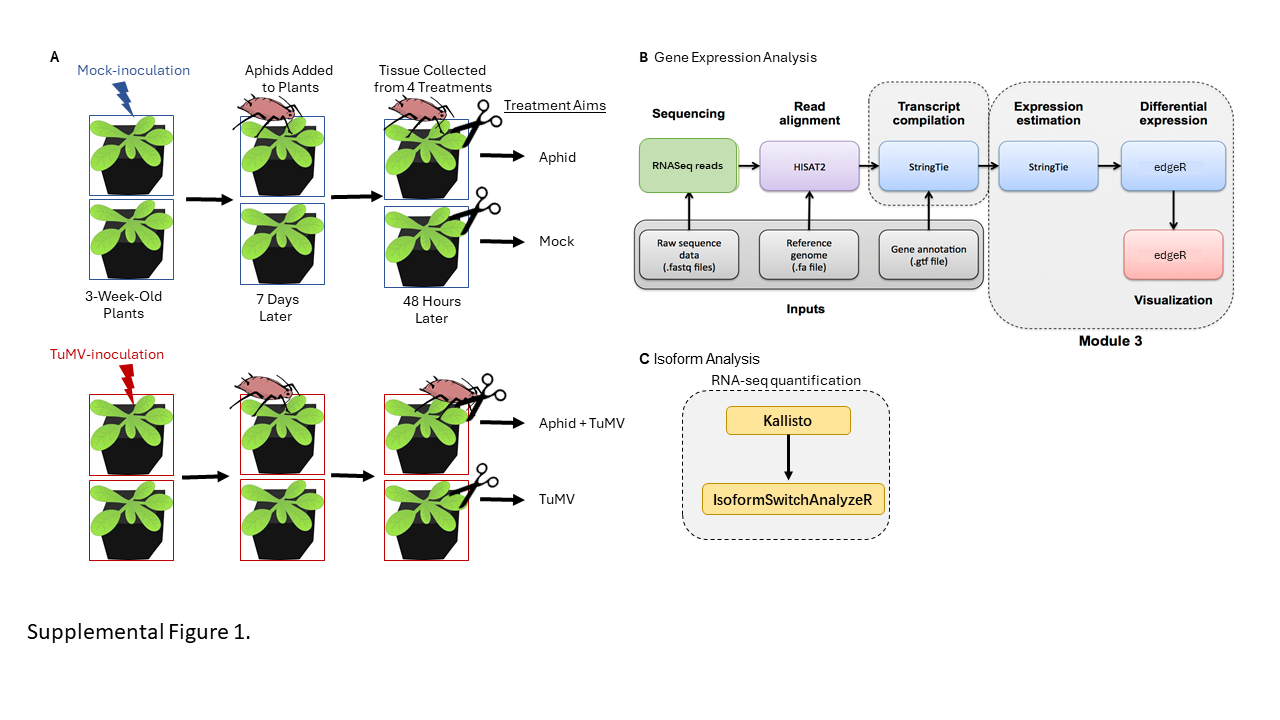

Supplement: Supplementary file 1 — Supplementary Material 1. [file 12870_2024_6014_MOESM1_ESM.tif]
