# Supplementary material for: Comprehensive transcriptomic analysis reveals turnip mosaic virus infection and its aphid vector Myzus persicae cause large changes in gene regulatory networks and co-transcription of alternative spliced mRNAs in Arabidopsis thaliana
Source: BMC Plant Biol. 2025 Jan 30;25:128. doi: 10.1186/s12870-024-06014-3 (PMC11780806; doi:10.1186/s12870-024-06014-3)
Supplement: Supplementary file 3 — Supplementary Material 3. [file 12870_2024_6014_MOESM3_ESM.pdf]

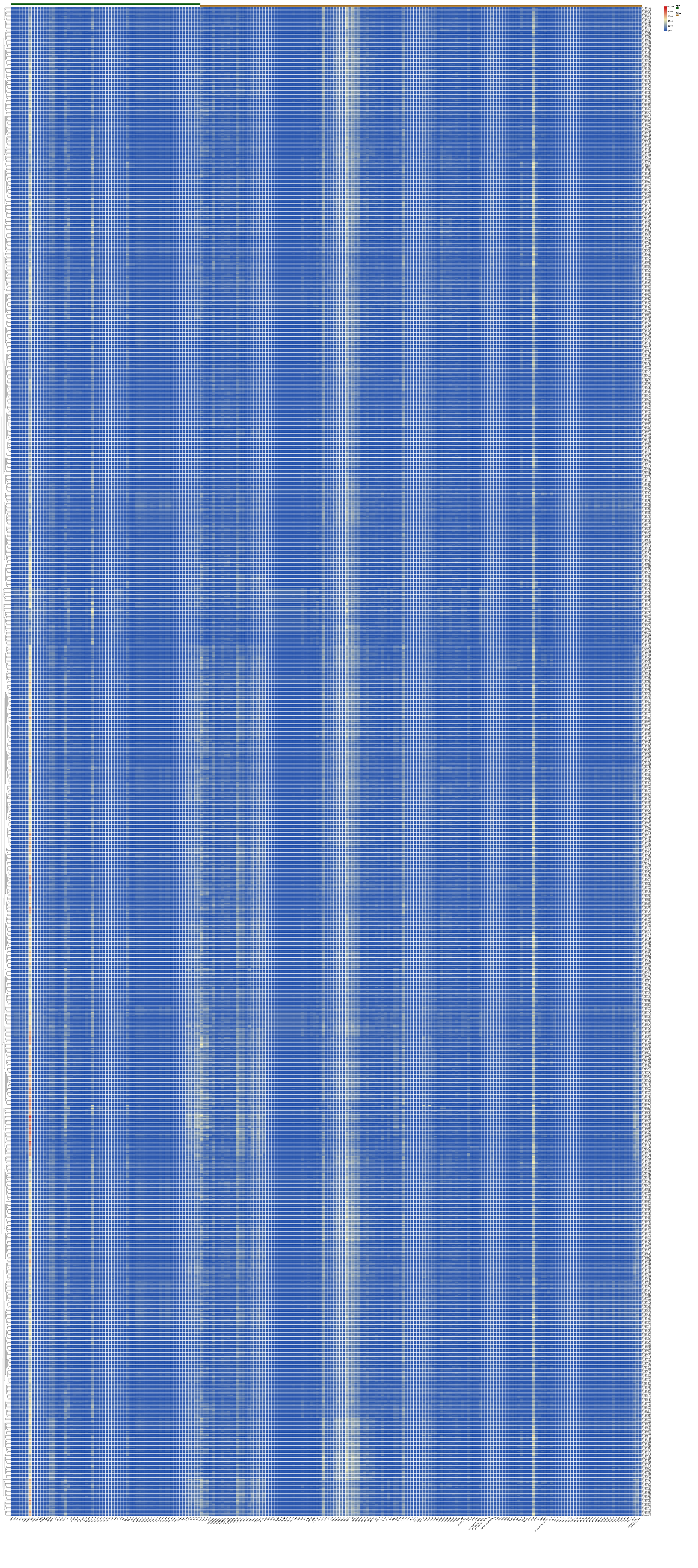
100.00
80.00
60.00
40.00
20.00
0.00
ABA
Other
